# Thrombosis of Bioprosthetic Aortic Valve: Is the Entire Arsenal Deployed?

**DOI:** 10.31083/j.rcm2507248

**Published:** 2024-07-05

**Authors:** Claudia Maria Loardi, Marco Zanobini, Emmanuelle Vermes, Maria Elisabetta Mancini, Anne Bernard, Christophe Tribouilloy

**Affiliations:** ^1^Department of Cardiac Surgery, Tours University Hospital, 37044 Tours, France; ^2^Department of Cardiovascular Surgery, Centro Cardiologico Monzino IRCCS, 20138 Milan, Italy; ^3^Department of Cardiology, Amiens University Hospital, 80054 Amiens, France; ^4^Department of Cardiovascular Imaging, Centro Cardiologico Monzino IRCCS, 20138 Milan, Italy; ^5^Department of Cardiology, Tours University Hospital, 37044 Tours, France

**Keywords:** bioprosthesis, aortic valve, thrombosis, echocardiography, computed tomography

## Abstract

The proliferation of transcatheter aortic valve implantation has alerted clinicians to a specific type of prosthetic degeneration represented by thrombosis. The pathogenesis of this clinical or subclinical phenomenon, which can occur in up to 15% of both surgical and percutaneous procedures, is poorly understood, as is its potential impact on patient prognosis and long-term bioprosthesis durability. Based on this lack of knowledge about the real meaning and importance of bioprosthetic valve thrombosis, the aim of the present review is to draw the clinicians’ attention to its existence, starting from the description of predisposing factors that may require a closer follow-up in such categories of patients, to an in-depth overview of all available imaging modalities with their respective pros and cons. Finally, a glimpse into the future of technology and biomarker development is presented. The hope is to increase the rate of bioprosthetic diagnosis, especially of the subclinical one, in order to understand (thanks to a strict and prolonged follow-up) if it can only be considered as an incidental tomographic entity without significant clinical consequences, or, on the contrary, if it is associated with neurological events or accelerated bioprosthetic degeneration. Nevertheless, despite the technical advances of echocardiography and cardiac tomography in terms of accurate bioprosthesis thrombosis detection, several diagnostic and therapeutic issues remain unresolved, including possible prevention strategies, tailored treatment protocols, and follow-up modalities.

## 1. Introduction

Bioprosthetic aortic valves have been surgically implanted for several decades, with the advantage of not requiring anticoagulants. Over the past ten years, their hegemony has gradually declined allowing the progressive spread of transcatheter aortic valve implantation (TAVI). Both prostheses face the problem of limited life span, especially in young patients, and the risk of valve degeneration, failure and thrombosis, a phenomenon that has attracted the interest of clinicians.

There are two distinct types of bioprosthetic valve thrombosis (BPVT), involving both surgical and percutaneously implanted bioprosthesis: clinically manifested thrombosis is characterized by a sudden increase in the transvalvular gradient with new-onset symptoms. Its incidence is estimated between 0.5–1.5% in surgically implanted bioprostheses whereas it occurs in 0.6–2.8% of cases after TAVI [1]. In contrast, subclinical leaflet thrombosis (LT) is an imaging finding revealed by computed tomography (CT) as hypoattenuated leaflet thickening (HALT) and restricted leaflet motion (RLM). It appears to be more common in TAVI patients than in surgical ones during the early follow-up period, whereas later their respective incidences become equal [2]. This entity is asymptomatic, although it may sometimes be associated with a slight increase in the transvalvular gradient [3] in the absence of true obstruction.

There are no clear guidelines addressing which protocol of follow-up for both surgical and percutaneously implanted patients must be applied to eliminate a subclinical LT: if everyone agrees and knows that a transthoracic echocardiography (TTE) is mandatory and easy to perform for a standard monitoring or in case of symptoms appearance, little is known about the need of adding another imaging modality (such as cardiac CT or transesophageal echocardiography, TEE) to screen for subclinical LT. In this perspective, the review begins by summarizing some predisposing factors to thrombosis that may help in identifying patients worthy of more stringent follow-up. Then, the different imaging modalities are described, with a special focus on cardiac CT including its possible application in asymptomatic thrombosis detection, findings, and technical issues.

It is suggested that a prompt recourse or standardized application of cardiac CT in high-risk patients should be proposed as a routine approach, especially considering that the impact of asymptomatic BPVT on early valvular dysfunction, increased thromboembolic risk, and consequently on patients’ neurological morbidity and mortality is still under investigation. Increased and more accurate detection of subclinical LT, with a subsequent appropriate follow-up, should allow to highlight its real clinical importance.

## 2. Predisposing Factors

Many characteristics are associated with LT, especially in the TAVI subgroup [4].

Concerning patient-related predictors, in patients implanted with a Sapien XT or Sapien 3 (Edwards Lifesciences, Irvine, CA, USA) male sex is associated with an increased incidence of subclinical LT, likely due to larger implanted prostheses and greater sinuses of Valsalva (two factors correlating with blood retention) [5].

In the same TAVI types, comorbidities also play an important role [4]:

- Obesity (body mass index >30 ${\mathrm{kg/m}}^{2}$) results in chronic inflammation and animbalance between pro- and antithrombotic molecules, leading to an increased risk of subclinical LT;

- Hypertension is associated with a gradual decrease in cardiac output with a reduced flow through the aortic valve;

- Chronic obstructive pulmonary disease and a smoking history imply a hypercoagulable situation, which is explained by constant bronchial and systemic inflammation and platelet membrane changes due to prolonged hypoxia;

- Chronic renal disease;

- In the PARTNER 3 trial, including balloon-expandable valves compared with standard surgical aortic valve replacement, rheologic factors affecting the Virchow triad, such as hypercoagulability secondary to factor V Leiden or prothrombin gene mutation, oral contraceptives, eosinophilia, and heparin-induced thrombocytopenia, represent an important risk factor for BPVT development [2].

Conversely, in patients undergoing TAVI both with balloon- or self-expandable devices, correctly anticoagulated atrial fibrillation (AF) is a protective feature [6]. The probable reason can be identified in the effect of oral anticoagulation (which is also the accepted treatment for both clinical and subclinical LT) in preventing thrombi formation or in dissolving initial blood clots on the leaflets.

Interestingly, no remarkable differences in the incidence of BPVT after TAVI with self-, balloon-, and mechanically expanding prostheses were found when comparing native bicuspid or tricuspid valves [7].

TAVI-related predictors of LT found in both self-expandable and balloon-expandable prostheses, include under-expansion and asymmetrical implantation, large-diameter prosthesis, oversizing by more than 20%, paravalvular leak, supra-annular implantation, valve-in-valve procedures and balloon-expandable devices: the lowest common denominator is an increase in blood stasis, major tissue damage, and local hemodynamic derangement [8]. Concerning the deployment modality, results from the RESOLVE/SAVORY registries suggest that the difference in LT rates between valve types correlates with the supra-annular versus intra-annular design, rather than the TAVI type [9], although intra-annular deployment, typical of balloon-expandable devices, was shown to be an independent predictor in a recent meta-analysis [10].

In addition, various blood-based biomarkers appear to be correlated with thrombus formation: if von Willebrand factor, thrombin-antithrombin complex, plasmin-α2-antiplasmin complex, prothrombin activation fragment 1+2 and D-dimer failed to prove their association with LT [11], N-terminal pro b-type natriuretic peptide (NT-proBNP) could potentially be used to monitor thrombosis regression during anticoagulant treatment in both balloon- and self-expandable TAVI thrombosis [12].

## 3. Clinical Presentation 

Depending on the time span after valve implantation, clinical BPVT may be acute (first 3 days), subacute (first 3 months, consisting of different layers of thrombus stratification) or chronic (over 1 year) [5]. Therefore, the clinical picture varies depending on the location, size, hemodynamic effects, and degree of valve obstruction. On the one hand, non-obstructive BPVT can remain asymptomatic and be discovered incidentally, while on the other hand, prosthesis-related significant thrombosis may lead to symptoms related to valve obstruction (e.g., dyspnea on exertion) or embolic complications [1, 13]. AF, left ventricular systolic dysfunction, and a hypercoagulable state increase the risk of systemic and pulmonary embolic phenomena [9].

Clinical examination may be deceiving, as stenotic murmurs can be subdued, and diagnosis relies mainly on imaging.

## 4. Transthoracic Echocardiography 

TTE is the mainstay triage tool in the diagnosis of clinical obstructive BVPT. Egbe *et al*. [14] demonstrated that the simultaneous presence of three criteria (an increased gradient >50% from baseline within the first five years after implantation in the absence of high cardiac output, increased cusp thickness, and abnormal mobility) appears to characterize BVPT with acceptable sensitivity and high specificity, yielding an area under the curve (AUC) of 0.852. More specifically, these echocardiographic findings, paroxysmal AF, and subtherapeutic international normalised ratio (INR) were strongly associated with BPVT, whereas moderate or more severe regurgitation was a rare finding compared to patients with structural valve failure. The detection of a layered thrombus in the prosthetic cusps on the downstream (arterial) side, is highly suspicious [15].

The Valve Academic Research Consortium 3 statement suggests specific thresholds for aortic prosthesis evaluation [16]: a gradient increase >10 mmHg or a gradient >20 mmHg should be considered abnormal and raise the possibility of BVPT.

Interestingly, the study by Naser *et al*. [17] highlighted that the mean gradients (MG) begin to rise months before the formal diagnosis of BPVT, recognizing that mildly abnormal gradients may be an early sign of subclinical thrombosis requiring closer monitoring. The study population included both surgical (porcine and pericardial) bioprosthesis as well as TAVI. Such a finding confirms that the immobilization of a prosthetic leaflet results in only a slight increase in MG, as showed by Makkar *et al*. [18] in a cohort of patients implanted with Portico (St Jude, Aboott, MN, USA), Sapien XT, and CoreValve (Medtronic Inc., Minneapolis, MN, USA) valves.

Recently, the New Mayo Clinic Algorithm [19] has been proposed to increase the sensitivity in detecting prosthetic valve obstruction: the combination of a Doppler velocity index <0.25, an abnormal appearance of valve cups (increased thickness, decreased mobility), and a decrease >20% in Effective Orifice Area from baseline resulted in an accuracy of 0.88 in diagnosing obstruction and correctly identified BPVT in 95% of patients.

Preexisting conditions such as low left ventricular ejection fraction, mild paravalvular leak and low-flow/low-gradient aortic stenosis increase the risk of developing LT. This underscores the importance of hemodynamic stasis on the valves and the need for a comprehensive clinical assessment in addition to echocardiographic parameters [20].

TTE is also central in monitoring the response to anticoagulation after BPVT diagnosis: usually, recovery is defined as a decrease of MG to the baseline, ≥50% compared with BPVT diagnosis or to the normal range depending on the model and size as well as resolution of valve thickening or restricted mobility. It appears that pericardial aortic valves recover more slowly than porcine ones, suggesting that a longer duration of warfarin might be required in the former category [17].

Conversely, the diagnostic accuracy of TTE in detecting subclinical nonobstructive BPVT is very limited: although it may be associated with a small increase in transvalvular gradient, this increase is often within the expected range for the type of bioprosthesis implanted [3].

## 5. Transoesophageal Echocardiography (TOE)

Current European Guidelines recommend TTE evaluation within 30 days of TAVI/surgical prosthesis implantation, after 1 year and then annually, with earlier follow-up in case of new symptom development [21]. However, acoustic shadowing by the device may preclude adequate visualization of the cusps, so TTE may not be sensitive enough to detect early hemodynamic changes in BPVT. Moreover, the diagnostic accuracy of TTE is influenced by other conditions, such as the presence of pericardial effusion, emphysema, obesity, or previous sternotomy. In these cases, TOE may be very useful because it has comparable sensitivity to CT for detecting thickening or restricted valvular mobility, as well as thrombotic appositions, even in the absence of symptoms or of increased transvalvular gradients.

Despite its greater invasiveness, it must be considered when TTE is suboptimal and in patients at increased risk of iodine-induced nephropathy. A deeper TOE longitudinal view with slight anterior flexion of the probe and a combination of multiple windows are recommended to avoid acoustic shadows [22]. The examination is more powerful for mitral and tricuspid than aortic BPVT diagnosis and it is very challenging in cases of valve-in-valve prostheses.

Nevertheless, even though TOE is superior to TTE in evaluating prosthetic dysfunction regardless of valve type, its diagnostic accuracy is greater for mechanical valves and it cannot reliably distinguish between BPVT and fibrotic pannus ingrowth. Although larger total mass volume and area, higher lesion density, more frequent abnormalities located on the aortic side, and greater limitation of valve motion are suggestive of thrombosis, the distinction between these two entities remains difficult [23].

## 6. Computed Tomography (CT)

The utility of CT in bioprosthesis dysfunction has risen rapidly over the past decade. Technological advances with wide-detector and dual-source scanners provide broader coverage and faster acquisition times, allowing for detailed morphologic and functional evaluation of the most commonly used surgical and TAVI valves, thus complementing other imaging modalities in identifying the underlying causes of prosthetic failure and guiding the most appropriate treatment [24].

**Fig. 1.**
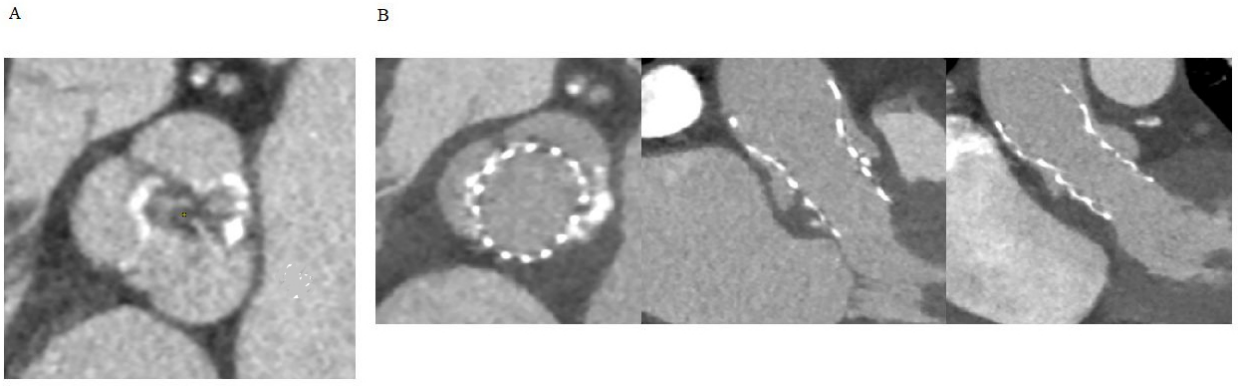
**Native bicuspid valve experiencing TAVI thrombosis.** (A) Axial view of the native valve showing bicuspid morphology with calcific raphe between the coronary cusps. (B) Axial, sagittal oblique and coronal oblique view of the transcatheter heart valve displaying post-procedural thrombosis of the non-coronary sinus. TAVI, transcatheter aortic valve implantation.

CT is not only crucial for the overall planning of TAVI, but can also be helpful in identifying specific factors that may be highly predictive of post-procedural thrombotic events: calcified tissue deposits or a bicuspid configuration that may alter the geometry and expansion of the TAVI, a particular composition of the damaged native valve that induces thrombosis due to exposure of tissue factor, and quantitative features of peri-aortic adipose tissue that are associated with an increased risk of LT [4] (Fig. 1).

CT is not routinely performed, but it must be considered when TTE and TOE are equivocal or limited by an inadequate acoustic window and there is a high clinical suggestion of prosthesis clinical thrombosis.

Généreux *et al*. [16] suggest that, after TAVI, prompt recourse to CT is mandatory in patients with significant echocardiographic valve deterioration defined as one or more of the following criteria: (1) transvalvular gradient of ≥20 mmHg and increase of ≥10 mmHg from baseline; (2) reduction of Doppler valve index of ≥0.1; and (3) new moderate-severe valvular regurgitation.

Some teams [25] prefer to avoid TOE and perform CT as the only imaging to confirm the diagnosis and to monitor patients under treatment. In their experience, one-third of patients with clinical or echocardiographic suspicion of LT were found to have evidence of LT on CT.

The technical approach is based on a triphasic acquisition. In the absence of contraindications to β-blockers, current heart rate control strategies should be considered to improve the visualization of bioprosthetic leaflets. Retrospective electrocardiographic (ECG) gating is essential for dynamic four-dimensional evaluation of valve mobility throughout the cardiac cycle, similar to cinefluoroscopy. In order to reduce the occurrence of metallic prostheses-related artifacts, acquisition at high tube voltages (120–140 kV) is preferred. The inclusion of a non-contrast-enhanced scan serves a dual purpose: distinguishing between suture material-like pledgets and paravalvular leak and evaluating the calcification of prosthetic heart valves, while a delayed phase (60–90 seconds) helps in recognizing thrombi and perivalvular complications, such as abscesses and pseudoaneurysms [26].

Differentiating thrombus from pannus relies on the assessment of tissue morphology and density. Pannus typically manifests as infiltration from beneath the sewing ring, extending towards the base of the leaflets and restricting their movement. Thus, it exhibits heterogeneous CT density, featuring regions of calcification and contrast enhancement due to the development of a microvascular network within the fibrotic material, with a cut-off level ≥145 hounsfield units (HU), similar to the interventricular septum. In contrast, thrombus is characterized by CT attenuation levels lower than those observed within the myocardium (≤90 HU), reflecting its different material composition, and manifests as an irregularly-shaped mass that adheres to either the leaflet or the hinge point [27]. It should be recognized that thrombus and pannus may coexist due to low flow states, consequent to gradual pannus formation.

Concerning subclinical LT, CT plays a key role to the point where it can be affirmed that the credit for the discovery of HALT and RLM phenomena with their relevant incidence belongs to this imaging modality. HALT refers to the presence of abnormal leaflet thickening associated with hypoattenuated material occurring early after TAVI and was first described by Pache in an 86-year-old man seven days after the implantation of a 29 mm sapien valve [28].

**Fig. 2.**
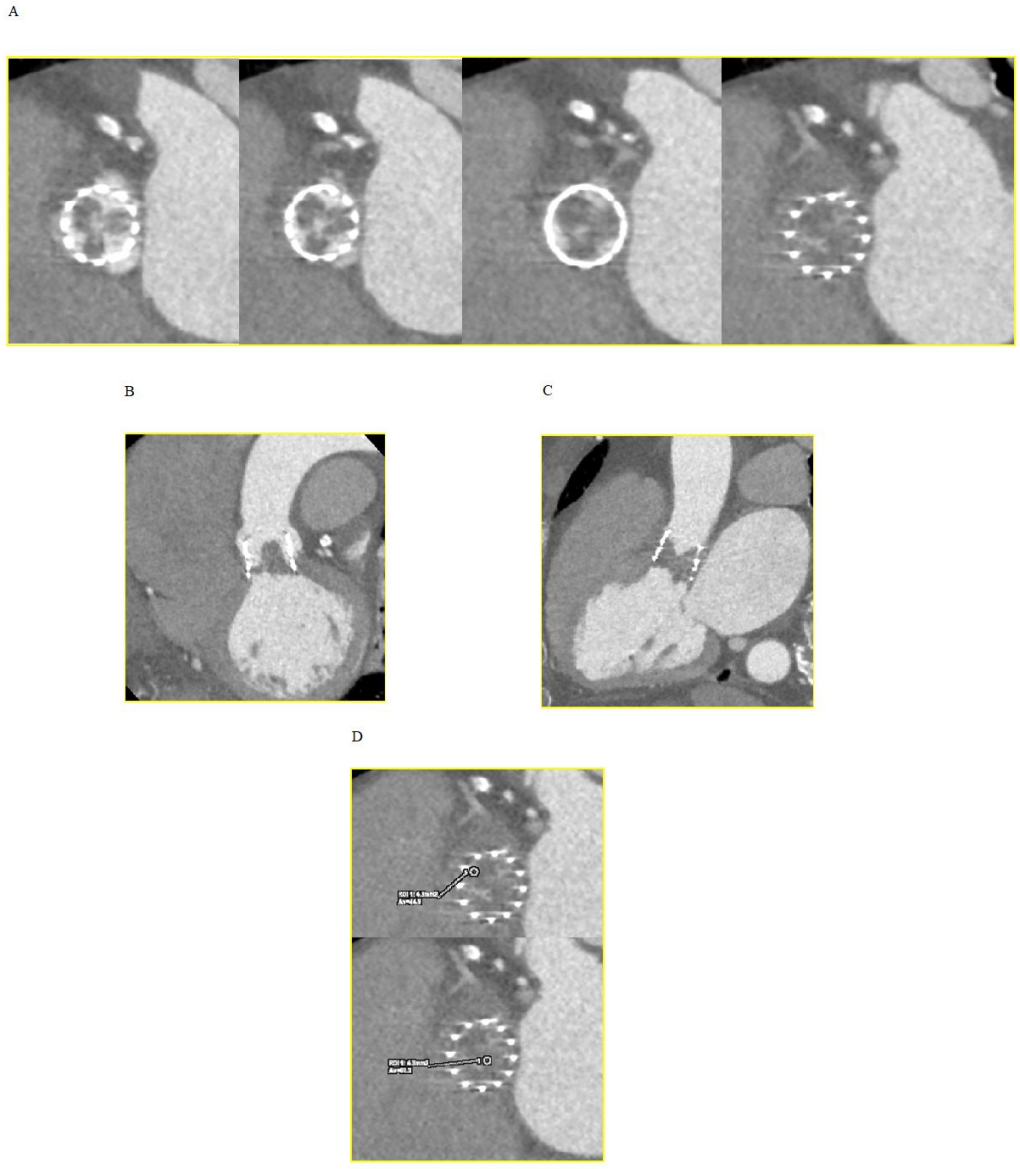
**Hypoattenuated lesions at CT.** (A) Short axis view of the transcatheter heart valve showing thickened and hypoattenuating leaflets. (B) Coronal oblique view. (C) Sagittal oblique view. (D) CT attenuation levels lower than those observed within the myocardium (≤90 HU) suggesting a thrombus. CT, computed tomography; HU, hounsfield units.

It is believed to be associated with localized thrombogenesis driven by activation of coagulation factors and perturbations in hemodynamics. Hypoattenuating lesions can be observed using multiplanar and three-dimensional (3D) volume-rendered CT reconstructions as 1–5 mm wedge-shaped or semi-lunar hypodense opacities, remaining visible during both systole and diastole (Fig. 2). They are typically located at the periphery and base of the leaflets and may extend to varying degrees to the edges of the leaflet in the center of the bioprosthetic frame, with the potential to result in RLM [29].

Valve leaflet motion is currently assessed at maximal leaflet opening during systole using a four-dimensional (4D) volume-rendered en-face image of the prosthetic valve, allowing for categorization as normal, slightly reduced (<50%), moderately reduced (50–70%), severely reduced (>70%), or immobile [29]. Because leaflet thickening originates at the base of the leaflet and extends to the tip, an imaging frame with maximal excursion is identified and the distance between the stent margin and the open leaflet tip is determined (Width, W); the distance between the stent frame margin and its center is the half of the distance (1/2D). The percentage reduction in leaflet motion is calculated as follows:

$$\%RELM=\frac{W}{\frac{1}{2\,D}}\times 100$$

Given the excellent CT spatial resolution, HALT has emerged as a more reproducible measure of subclinical LT, whereas RLM, which is more technically demanding and depends on the modest temporal resolution of CT compared to TTE, should only be evaluated in the context of HALT to avoid overdiagnosis [30].

The precise comprehension of the natural course of this condition remains somewhat limited, and although subclinical LT may not present immediate clinical consequences, there is a notable concern regarding potential embolic complications or premature prosthesis degeneration.

Blanke *et al*. [31] found approximately 17% of HALT at 30 days and 30% at 1 year follow-up and a frequency of RLM of 14% and 29% respectively, with no significant difference between TAVI and surgical prostheses. In their study, the authors considered the Evolut TAVI (sizes from 23 to 34) and different types of surgical pericardial bioprosthesis (sizes 21–27). However, when considering the extent of HALT severity on the affected leaflet, the percentage was mostly less than 25% in the TAVI group, whereas it was more often (25–50%) in the surgical group. A largely immobile leaflet occurred only when the extent of thickening was greater than 75% on the leaflet. These findings did not result in significant echocardiographic changes or different adverse outcomes, although consensus on this point is far from unanimous [29, 32, 33]. For instance, the PARTNER 3 study showed that patients with HALT had a significantly higher MG and a trend toward a higher composite end-point of stroke/transient ischemic attack and thromboembolic complications at 1 year [2].

Aside from leaflet evaluation, CT allows accurate geomorphological assessment of the TAVI device, such as prosthesis asymmetry, expansion, and depth [34]. A recent observational study demonstrated an association between prosthesis under-expansion and depth with the development of LT [35]. Post-procedural CT imaging also enables for the evaluation of valve alignment, which may affect valve hemodynamics and future LT [34].

Dual or single antiplatelet therapy appears to have limited efficacy in the prevention and management of subclinical LT. However, emerging evidence suggests that oral anticoagulants may have an encouraging role in alleviating these concerns, either through protective or therapeutic mechanisms [36].

## 7. Specifics of Valve-in-valve Procedures

Clinical or subclinical LT may also occur after transcatheter valve-in-valve implantation, with the potentially catastrophic consequence of the extension of thrombosis to the coronary network. The International Registry by Abdel-Wahab *et al*. [37] is the largest available study addressing this issue: it showed that the incidence of clinical thrombosis, diagnosed after a median time of 101 days based on a combination of new-onset valve dysfunction and imaging evidence of LT, reached 7.6% of cases. Associated factors included the absence of oral anticoagulation, the true internal diameter of the surgical valve indexed to body surface area and a stented porcine bioprosthesis. Interestingly, there were no deaths, strokes, or myocardial infarctions related to valvular thrombosis.

With the goal of reducing the risk of post-procedural valve-in-valve thrombosis, leaflet laceration with the BASILICA technique appears to be able to mitigate neo-sinus and sinus flow stasis by improving washout [38].

Application of the promising new tool of numerical fluid dynamics simulations in this particular area suggests that two lacerations provide the best results in terms of reduction of a high-risk area for thrombi formation [39].

## 8. Future Perspectives

### 8.1 Laboratory Markers

One promising line of research focuses on the potential role of LT biomarkers represented by platelet extracellular vesicles [40, 41]. These are nanoparticles released by platelets that may serve as modulators of inflammation, vascular dysfunction, and thrombosis. Notably, the TAVI procedure has been shown to modulate their composition in the bloodstream by decreasing the concentration of platelet vesicles and increasing the concentration of endothelial cell-derived vesicles. Their variety, content, and functions hold promise as possible specific molecules for LT.

### 8.2 Imaging and Simulations Predictors of TAVI Thrombosis Risk

Although device-related procedural difficulties are known to correlate with the development of BPVT, it is difficult to identify accurate CT-imaging predictors of a potentially suboptimal TAVI outcome. Coupling CT-imaging with finite element analysis [42] may allow the creation of a biomechanical model of the patient’s aortic root and leaflets; this could predict the presence of calcified refractory blocks, the deformation or incomplete expansion of the prosthetic stent, and the development of a paravalvular orifice possibly leading to TAVI thrombosis.

### 8.3 New CT Paradigms

In addition, the radiomic approach can greatly increase the amount of quantitative information that can be obtained from CT images by extracting multiple imaging features that are indiscernible to the human eye [43]. Radiomics uses texture analysis to model the spatial distribution of voxel greyscale intensities, applies statistics to provide a measure of heterogeneity and quantifies the shape and size of three-dimensional volumes within an imaging dataset, resulting in large data patterns potentially associated with LT [44].

### 8.4 Positron Emission Tomography (PET)-CT

An emerging helpful tool is represented by PET-CT using the glycoprotein IIb/IIIa receptor radiotracer 18-fluorine glycoprotein 1 (18F-GP1). In *ex-vivo* experiments on human platelets and explanted bioprosthetic valves, Bing *et al*. [45] showed that, although adherence of activated platelets to bioprosthesis is common, increased marker uptake was independently associated with the presence of HALT and correlated with regression of thrombosis in patients treated with anticoagulation. Furthermore, no uptake was observed in areas of fibrosis, suggesting that 18F-GP1 may differentiate thrombus from fibrosis. Nevertheless, no thresholds for “normal” or pathological uptake can be established at present. Since complete agreement between TTE and PET-CT thrombosis diagnosis has not been found, their respective roles, accuracy and interrelationship need to be comprehensively clinically interpreted and further investigated.

## 9. Treatment and Outcomes

Once the diagnosis of clinical BPVT is established, oral anticoagulation treatment is initiated [1, 46]. Current guidelines recommend vitamin K-antagonists or unfractionated heparin in hemodynamically stable patients. In case of refractory hemodynamic impairment and in the absence of contraindications, urgent surgical intervention may be proposed. Alternatively, fibrinolytic therapy should be considered in unstable patients who are not surgical candidates.

In clinical BPVT, oral anticoagulation is effective in approximately 90% of cases, with a significant decrease in MG or resolution of the thrombotic mass within two months of treatment. Only a small percentage of patients require repeat valve procedures [47].

Regarding subclinical LT, it is recognized as a dynamic process, as clearly demonstrated in the Evolut Low Risk trial [31], where, among the 17% of patients with HALT at 30 days, 36% had spontaneous resolution at 1 year, and 23% had a spontaneous appearance of HALT at 1 year who did not have HALT at 30 days. Similar data were reported in the PARTNER 3 cardiac CT substudy [2]. Despite this variable and transient feature of the natural history of subclinical LT, treatment with oral anticoagulation is recommended, as its efficacity has been proven with a complete resolution of HALT in almost all treated patients [9].

Whether to continue oral anticoagulation long-term after successful treatment of an initial episode of clinical or subclinical LT is another subject of debate, and should be based on individual assessment, also considering that recurrence of BPVT has been reported warranting long-term anticoagulation regime [48].

## 10. Gap of Evidence

Several aspects of subclinical LT still remain unclear, with conflicting evidence emerging from the medical literature. In particular, its possible relationship with patient prognosis is an intriguing but unresolved issue: regarding the domain of neurological events, Hein *et al*. [49] affirm the lack of an association with an increased rate of cerebrovascular accidents or mortality at mid-term follow-up, a finding in complete contrast with a recent meta-analysis showing an increased incidence of stroke [11]. Similarly, the question of whether HALT and RLM are predictors of future premature valve degeneration is very controversial: on the one hand, Rashid *et al*. [50] clearly demonstrate a strong association between these two phenomena by indicating specific depth and area thresholds on CT, on the other hand, several observational studies [2], a randomized controlled trial [51], and a meta-analysis [52] fail to undoubtedly reach the same conclusion, describing comparable or at most only slightly increased transvalvular gradients at 6 months and 1 year in patients with LT.

All of this controversy is reflected in the lack of guidelines addressing the optimal treatment to prevent thrombosis after TAVI or surgery (warfarin, dual or single antiplatelet therapy, or no anti-thrombotic therapy at all) [53, 54, 55] as well as the duration of anticoagulation treatment after LT diagnosis. Also, the need and timing of monitoring the evolution of HALT with routine multidetector CT remain uncertain and are worthy of further investigation [56].

## 11. Conclusions

While clinical BPVT poses no real diagnostic problems and is quite rare, subclinical LT represents a mysterious entity, whose detection is often difficult and doubtful and requires a multimodality diagnosis.

In case of symptom appearance and consequent suspicion of clinical thrombosis, TTE is recognized as the gold standard diagnostic tool. On the contrary, subclinical LT is, by definition, not symptom-driven, TTE is of little contribution and only CT can detect it with certainty. Nevertheless, the modalities of performing a control CT are unknown: should every patient receiving a surgical bioprosthesis or a TAVI be screened? And with what temporal pattern? Perhaps an aggressive and tailored follow-up protocol, including a CT control, should be applied when multiple risk factors are present, such as a hypercoagulable state coupled with certain TAVI-related features favoring thrombosis.

The subsequent increased attention to subclinical LT diagnosis will allow clarification of its clinical relevance, as there is still no certainty that it is not related to mid- or long-term bioprosthesis failure or rapid degeneration. It is also conceivable that there is a continuum between the mere imaging finding of a thrombus and the actual development of obstruction or symptoms, but this phenomenon is far from being fully understood.

Since the discovery of subclinical LT coincides with the birth of TAVI, its “existence” is brief and no data are available on its impact on daily life. In this uncertain context, it is imperative to use all possible diagnostic tools to detect the problem, starting from the identification of predisposing factors that may lead to more accurate patient follow-up until the monitoring of progressive resolution in case of initiation of anticoagulant treatment.

In this regard, the validation of early markers, including circulating extracellular vesicles or new imaging protocols (CT or PET-CT), can be very interesting but requires endorsement in larger trials, also considering that the findings of these different imaging modalities are difficult to interpret and sometimes do not match each other.

Concerning the possible relationship between clinical and, in particular, subclinical BPVT and prognosis, it is hard to imagine that a CT-detected RLM does not imply a disruption or at least an upheaval of the structure of the cusp without future clinical consequences. The data are conflicting, but, since the doubt of accelerated bioprosthetic failure in overt BPVT and of the association between HALT and embolic events persists, clinicians should continue their efforts to improve and tailor the diagnosis and perhaps consider temporary prophylactic anticoagulation treatment in high-risk patients to prevent possible complications.
